# Evaluation of the chemical defense fluids of *Macrotermes carbonarius* and *Globitermes sulphureus* as possible household repellents and insecticides

**DOI:** 10.1038/s41598-020-80018-5

**Published:** 2021-01-08

**Authors:** S. Appalasamy, M. H. Alia Diyana, N. Arumugam, J. G. Boon

**Affiliations:** 1 Institute of Food Security and Sustainable Agriculture (IFSSA), Universiti Malaysia Kelantan, Jeli Campus, 17600 Jeli, Kelantan Malaysia; 2 Faculty of Earth Science, Universiti Malaysia Kelantan, Jeli Campus, 17600 Jeli, Kelantan Malaysia; 3 Faculty of Bioengineering and Technology, Universiti Malaysia Kelantan, Jeli Campus, 17600 Jeli, Kelantan Malaysia

**Keywords:** Ecology, Zoology

## Abstract

The use of chemical insecticides has had many adverse effects. This study reports a novel perspective on the application of insect-based compounds to repel and eradicate other insects in a controlled environment. In this work, defense fluid was shown to be a repellent and insecticide against termites and cockroaches and was analyzed using gas chromatography-mass spectrometry (GC–MS). *Globitermes sulphureus* extract at 20 mg/ml showed the highest repellency for seven days against *Macrotermes gilvus* and for thirty days against *Periplaneta americana*. In terms of toxicity, *G. sulphureus* extract had a low LC_50_ compared to *M. carbonarius* extract against *M. gilvus*. Gas chromatography–mass spectrometry analysis of the *M. carbonarius* extract indicated the presence of six insecticidal and two repellent compounds in the extract, whereas the *G. sulphureus* extract contained five insecticidal and three repellent compounds. The most obvious finding was that *G. sulphureus* defense fluid had higher potential as a natural repellent and termiticide than the *M. carbonarius* extract. Both defense fluids can play a role as alternatives in the search for new, sustainable, natural repellents and termiticides. Our results demonstrate the potential use of termite defense fluid for pest management, providing repellent and insecticidal activities comparable to those of other green repellent and termiticidal commercial products.

## Introduction

A termite infestation could be silent, but termites are known as destructive urban pests that cause structural damage by infesting wooden and timber structures, leading to economic loss. Despite the negative perception that humans have of termites as pests, termites play a vital role in the maintenance of soil organic matter in natural habitats and in agroecosystems^1–3^ in both forests and urban environments. Termites are also an important food source for various other insects owing to their vast abundance in a range of habitats, with their main predators being ants^4,5^. Upon facing such strong predation from ants, termites have evolved a defense system involving the presence of specialized soldier termites amid sterile ones. Termites have both mechanical and chemical defense mechanisms^4,6^. However, termite defense mechanisms are one of the least explored and reported subjects. This intriguing insect defense mechanism could reveal the interrelationship and coevolution of compounds and termite species. The earliest insect defense mechanism was reported in^4^, and various defense mechanisms in termites were listed, including sensory organs, physical defenses, and chemical secretion. However, for chemical secretion, only a few species of termites, such as *Macrotermes carbonarius* (Blattodea: Termitidae) and *Globitermes sulphureus,* and their chemical compounds of interest were reported as of June 2010^7^.

The termite defense fluid helps to protect their colony by secreting acetate-derived compounds (from fatty acid metabolism) found in *Macrotermes* and *Globitermes*^6^. In a related study, the terpenoid hydrocarbon *β*-selinene excreted by *Reticulitermes speratus* helped fend off the Asian needle ant *Brachyponera chinensis* and ponerine ant *Myopias amblyops*^8^. Similarly, *M. carbonarius* and *G. sulphureus* soldiers can defend themselves from other insects by secreting defense fluid^9,10^. In a previous study, termites from the family Mastotermitidae and several from the subfamily Macrotermitinae produced quinone mono- and sesquiterpenes with macrocyclic lactones that are toxic and irritant and have congealing effects on the secretion released into the wound^11^. This proves that the existence of chemical compounds in the defense fluid has a particular repellent and toxic effect on other insect species.

The introduction of insect-based products as natural pesticides in industry is especially common. These products utilize pheromones from semiochemicals produced via animal communication^12^. In Malaysia, pheromone traps are currently used as one of the products in the integrated pest management program for Lepidoptera and Coleoptera^13^. At present, chemical-based products are still in use, despite the introduction of biopesticides. The drawbacks of chemical-based products are their negative effects on safety, public health and the environment, including sublethal effects on nontarget species, such as the effects on the foraging patterns of honey bees^14,15^. In contrast, the production of plant-based pesticides is challenging. The utilization of essential oils as natural pesticides, such as oils from *Mentha* spp. and *Lavanda* spp. that help in the inhibition of acetylcholinesterase, is affected by the low quality, productivity and factor dependence (i.e., soil acidity) of these oils^16–18^. Thus, the essential oil composition can change along with changes in various factors.

Natural pesticide product development is more focused on agricultural pests than on urban pests because of the economic benefits. This can be identified through the trends of publications over the years regarding the biological control of termites^19^. For example, one of the studies described the lack of success in the biological control of termites, as *Nasutitermes* species have evolved to resist diseases using biochemical and immunological strategies^20^. Hence, this research was conducted to study the potential of termite defense fluids as natural pesticides by utilizing chemical communication. Defense fluids of *Macrotermes carbonarius* and *Globitermes sulphureus* were used in this study. These species were selected due to their high abundance in rural areas of Kelantan^10^. In addition, both species are known as defense fluid producers and consist of a high number of soldier castes that can be found in mounds or colonies^7^.

## Materials and method

### Ethics declaration

All applicable international and national guidelines for the care and use of animals were followed in this study. All procedures performed involving animals (insects) were conducted in accordance with the ethical standards of the institution or practice at which the studies were conducted.

### Species collection and identification

This study was conducted in Kelantan state, Malaysia. The soldier castes of *M. carbonarius* and *G. sulphureus* were collected from mounds around Rantau Panjang and Jeli, Kelantan. The specimens were brought back to the postgraduate laboratory of the Faculty of Earth Science, Universiti Malaysia Kelantan, Jeli Campus, and maintained at room temperature (23 °C) with relative humidity greater than 50% in dark conditions. Identification of the termite soldier morphology was performed according to keys provided in^21^ with the aid of a MOTIC 2500 5.0 MP Live Resolution (MOTIC, Hong Kong, China) camera attached to a stereomicroscope.

### Extraction of defense fluid

The defense fluid extraction protocol was adapted from^22^ with modification of the extraction duration. Soldier termites were rinsed with distilled water and then air-dried. Then, both species were dissected, weighed, homogenized and extracted in methanol for 24 h of extraction (HPLC grade; MERCK, Selangor, Malaysia). The extract was filtered by using a muslin cloth and WHATMAN Filter Paper No. 1 (SIGMA ALDRICH, Darmstadt, Germany). Then, the solution was dried using a rotary evaporator (IKA, Staufen, Germany) at 60 °C. The extract was weighed and stored at − 20 °C until further use.

### Repellency against the termite *Macrotermes gilvus*

This method was modified from^23^. The termite species *Macrotermes gilvus* was chosen to test the repellency of the extracted defense fluid. *Macrotermes gilvus* is the most abundant pest species that attacks the structural buildings in Kelantan^10^. A colony of *M. gilvus* was collected from mounds around Jeli, Kelantan. The boxes (28 L) were filled with a mixture of sand. The defense fluid extract was diluted to five concentrations using methanol (0, 1, 5, 15, and 20 mg/ml). The concentrations were used in every assay in this study. Half of the sand area was mixed with the extract. Thirty termite individuals (25 workers, 3 minor soldiers, 2 major soldiers) were released into the box^24^. The fungal combs were provided in small quantities (2 g), and hyphal growth was monitored and removed every 12 h. The positive control used in this study was a commercial natural insect repellent, WATSON Insect Repellent (WATSON, Hong Kong, China), due to the unavailability of termite chemical repellents. Each treatment was prepared in three replicates, and observations were recorded daily at 20:00 h. The repellency data were recorded for seven days for *M. gilvus.*

### Repellency against the cockroach *Periplaneta americana*

The repellency assay was modified from the protocol reported in^24,25^. Adult *Periplaneta americana* cockroaches were purchased from the Vector Control Research Unit, School of Biological Sciences, Universiti Sains Malaysia (USM), Penang Island. The repellency chamber was set up by using two boxes (45 L), and a 30 mm diameter acrylic tube was used as the connector. Both chambers were supplied with an egg carton for hiding, and cat food, water, and wood shavings were placed on the chamber floor. Food and water were provided ad libitum. All of the conditions were prepared according to^26^. Both chambers were covered using muslin cloths and secured with Velcro tape without a lid. The solution was applied on the surface of the treated box. Then, the treated surface was dried for 10 min. Ten cockroaches were released in both areas. The boxes were kept for 12 h under light conditions (approximately 71 lx) and 12 h under dark conditions. Commercial naphthalene (GANSO, Johor, Malaysia) was used as a positive control, and methanol was used as a negative control. The test was carried out for 30 days in triplicate, and data were recorded by opening the muslin cloth and observing the number of cockroaches in each chamber daily at 22:00 h.

### Toxicity against the termite *Macrotermes gilvus*

The method was modified from^23,24^ and tested on the termite *M. gilvus*. Filter paper was soaked with the extract at five concentrations. The fungal comb was not provided as it was a force-feeding-based design. The arena was supplied with a plate of water at 70–80% humidity. Triplicates were examined for every concentration, and the experiment was set up using a 7.5 ml box. The mortality data were recorded daily at 20:00 h. The commercial product Chlorpyrifos (DYNA-PEST, Kuala Lumpur, Malaysia) was used as a positive control, and methanol was used as a negative control.

### Toxicity against the cockroach *Periplaneta americana*

For the toxicity test of the cockroach *P. americana*, the method was adapted from^26,27^. Test cockroaches were starved for three days before the toxicity test. Five concentrations of extract were mixed with crushed cat food (POWERCAT, Selangor, Malaysia) and fed to the cockroaches. Commercial cockroach insecticide (BAYGON, Wisconsin, US) was diluted in methanol at 20 mg/ml and mixed with crushed cat food as a positive control. The data for cockroach mortality were recorded every hour on the first day and every 12 h subsequently for 30 days. The cockroaches were considered dead when no movement was observed after they were touched with forceps. The experiment was performed in triplicate.

### Compound separation

The compound separation procedure was divided into two parts: thin layer chromatography (TLC) and gas chromatography–mass spectrometry analysis (GC–MS). The extract was fractionated and analyzed according to^28^. Silica gel F_254_ MERCK (MERCK, Selangor, Malaysia) and two mobile phases were used. The mobile phases were chloroform/methanol/water (98:2:1, v/v/v) and hexane/diethyl ether/acetic acid (8/5/0.2, v/v/v). The fractioned components were visualized by charring the plate at 200 °C under a flame for *M. carbonarius* and viewing under 254 nm UV light in a UV Viewing Chamber (UVITEC, England, United Kingdom) for *G*. *sulphureus*. The standards used were lauric acid methyl ester (SIGMA-ALDRICH, Missouri, USA), pentadecanoic acid (SIGMA-ALDRICH, Missouri, USA), octadecanoic acid (SIGMA-ALDRICH, Missouri, USA), and tridecane (SIGMA-ALDRICH, Missouri, USA). The TLC results were used to visualize and preconfirm the presence of compounds. For GC–MS, termite defense fluid extracts were diluted and analyzed using GC–MS-QP2010 (SHIMADZU, Columbia, USA) with a SLB5MS column. The solvent delay was 2 min, injector temperature was 280 °C, MS quadrupole temperature was 150 °C, MS source temperature was 230 °C, and transfer line temperature was 300 °C, with splitless mode injection and a helium flow rate of 0.75 mL/min. The extracts were diluted in methanol and injected into the GC–MS instrument. The analytical standard was spiked at 1 ppm. The standards used were as described in the first part. Compound identification was performed using mass spectrometric detectors and the NIST17 MS library database.

### Data analysis

The repellent activity was corrected to the percentage of repellency (PR) using the formula described in^29^, as shown in Eq. (1). The PR was classified as in Table 1. Then, the PR and percentage of toxicity against time were analyzed by ANOVA according to days for the longevity of the extracts and Duncan’s test as a post hoc test at an alpha level of 0.05. The statistical analysis (ANOVA and Duncan’s test) was conducted using SPSS Statistics Version 23 (IBM, NY, US). The results are expressed as the mean ± standard deviation.

**Table 1:** Scale used to estimate the class for mean percentage repellency.

Class	Repellency rate (%)	Interpretation
0	> 0.01–< 0.1	Nonrepellent
I	0.1–20	Very weakly repellent
II	20.1–40	Moderately repellent
III	40.1–60	Average repellency
IV	60.1–80	Fairly repellent
V	80.1–100	Very repellent

1 $$PR=\frac{\left[Nc-Nt\right]}{\left[Nc+Nt\right]}\times 100$$

where Nc is the number of insects in the control group, and Nt is the number of insects in the treatment group.

## Result

### Repellency effect and efficiency against termites

*Globitermes sulphureus* produces a higher volume of defense fluid (2.93 g for 100 soldiers) than *M. carbonarius* (0.68 g for 100 soldiers). Almost all concentrations of the defense fluid extracts of *M. carbonarius* and *G. sulphureus* showed insect pest repellent activity, and the fluid was categorized as a class IV to class V repellent (Table 2). *Macrotermes carbonarius* extract at a concentration of 1 mg/ml showed the lowest mean percentage of repellency (68.57%) compared to *G. sulphureus* (83.05%)*.* The highest mean percentage repellency was observed for extracts of both species at 20 mg/ml, with values of 98.00% and 97.62%, respectively. At 0 mg/ml, the percentage of repellency was zero, and this value was used for data normalization for other concentrations.

**Table 2:** Mean percentage repellency with the standard error and repellency class of *Macrotermes carbonarius* and *Globitermes sulphureus* extracts against *Macrotermes gilvus*.

Day	Percentage of repellency (%)				
	1	5	15	20	Positive control
**Concentration of G. sulphureus extract (mg/ml)**					
1	93.33 ± 1.67^a^	91.67 ± 1.67^ab^	91.67 ± 1.67^b^	85.00 ± 2.87^c^	88.33 ± 1.67^e^
2	91.67 ± 1.67^a^	98.33 ± 1.67^b^	96.67 ± 3.33^b^	100.00 ± 0.00^c^	100 ± 0.00^e^
3	91.67 ± 1.67^a^	98.33 ± 1.67^b^	96.67 ± 3.33^c^	98.33 ± 1.67^d^	100 ± 0.00^e^
4	93.33 ± 1.67^a^	95.00 ± 2.89^b^	98.83 ± 1.67^c^	100.00 ± 0.00^d^	100 ± 0.00^e^
5	86.67 ± 1.67^a^	88.33 ± 1.67^ab^	98.83 ± 1.67^b^	100.00 ± 0.00^d^	100 ± 0.00^e^
6	38.33 ± ± 1.67^a^	80.00 ± 2.89^b^	91.67 ± 1.67^c^	100.00 ± 0.00^d^	100 ± 0.00^e^
7	31.67 ± 1.67^a^	78.33 ± 1.67^ab^	81.67 ± 1.67^b^	100.00 ± 0.00^c^	100 ± 0.00^d^
Mean PR (%)	83.05	90.48	93.81	97.62	98.33
Repellency Class	V	V	V	V	V
**Concentration of M. carbonarius extract (mg/ml)**					
1	56.00 ± 1.00^ab^	86.67 ± 1.67^b^	88.33 ± 1.67^c^	98.33 ± 1.67^d^	88.33 ± 1.67^e^
2	75.$67\pm$ 2.33^ab^	100.00 ± 0.00^b^	89.67 ± 0.33^c^	91.67 ± 1.67^d^	100 ± 0.00^e^
3	81.67 ± 1.67^ab^	88.33 ± 1.67^bc^	98.33 ± 1.67^c^	100.00 ± 0.00^d^	100 ± 0.00^e^
4	85.00 ± 2.89^ab^	93.33 ± 1.67^bc^	90.00 ± 2.89^c^	97.67 ± 1.45^d^	100 ± 0.00^e^
5	82.00 ± 1.52^ab^	96.67 ± 1.67^bc^	100.00 ± 0.00 ^c^	98.33 ± 1.67^d^	100 ± 0.00^e^
6	72.33 ± 1.45^ab^	88.33 ± 1.67^bc^	100.00 ± 0.00 ^c^	100.00 ± 0.00^d^	100 ± 0.00^e^
7	42.00 ± 1.53^a^	93.33 ± 1.67^ab^	93.33 ± 1.67^b^	100.00 ± 0.00^c^	100 ± 0.00 ^d^
Mean PR (%)	68.57	91.90	93.33	98.00	98.33
Repellency Class	IV	V	V	V	V

Different letters in the same row indicate a significant difference (*p* < 0.05, Duncan’s test).

Mean PR = mean percentage of repellency. Mean of three replicates (n = 30 termites per replicate).

Repellency class = class 0 (> 0.01 to < 0.1%), nonrepellent; class I (0.1 to 20%), very weakly repellent; class II (20.1–40%), moderately repellent; class III (40.1–60%), averagely repellency; class IV (60.1 to 80%), fairly repellent; class V (80.1–100%), very repellent.

Repellency activities per day observed for *M. carbonarius* and *G. sulphureus* extracts against pest termite *M. gilvus* are tabulated in Table 2. The minimum repellency of the *M. carbonarius* extract (1 mg/ml) was 42%, whereas for *G. sulphureus,* it was 31.67% after exposure to pest termites for seven days. Both extracts showed low repellent activity at the beginning of the experiment (day 1), and the repelling intensity increased from day 2 until day 4. The repellency of both extracts against *M. gilvus* began to decline from day 5 until day 7. However, both extracts at 15 and 20 mg/ml showed an increasing trend of repellency after four days of exposure, reaching 100.00% repellency against pest termites. These results were comparable to those of the positive control. Overall, these results indicated that even at low concentrations, *M. carbonarius* and *G. sulphureus* extracts showed repellency activities against pest termites.

### Repellency effect and efficiency on cockroach

The defense fluids of *M. carbonarius* and *G. sulphureus* soldier termites were tested on another pest, the American cockroach *P. americana* (Table 3). The repellent activity of *M. carbonarius* extract achieved only class II and III values (28.61% and 56.83%), including no repellence effect even at the highest concentration, 20 mg/ml. The highest percentage repellency (78.56%, class IV) of the *G. sulphureus* extract was observed at a concentration of 20 mg/ml.

**Table 3:** Mean percentage repellency with the standard error and repellency class of *Macrotermes carbonarius* and *Globitermes sulphureus* extracts against *Periplaneta americana*.

Day	Percentage of repellency (%)				
	1	5	15	20	Positive control
Concentration of *M. carbonarius* extract (mg/ml)					
5	− 19.33 ± 1.76^a^	26.67 ± 2.40^b^	49.33 ± 1.33^c^	14.67 ± 1.76^d^	44.00 ± 2.31^e^
10	7.33 ± 1.76^ab^	24.67 ± 1.76^b^	69.33 ± 2.40^c^	30.67 ± 1.33^d^	36.67 ± 2.44^e^
15	13.33 ± 2.67^a^	41.33 ± 1.33^b^	45.33 ± 1.33^c^	− 19.33 ± 1.33^d^	6.00 ± 1.15^e^
20	52.67 ± 1.76^a^	22.67 ± 2.67^b^	49.00 ± 2.52^c^	− 19.33 ± 0.67^d^	71.00 ± 2.51^e^
25	20.00 ± 2.31^a^	30.67 ± 1.20^b^	62.67 ± 2.67^c^	− 14.33 ± 0.88^d^	66.67 ± 3.53^e^
30	11.33 ± 2.40^ab^	22.67 ± 2.67^b^	65.33 ± 1.16^c^	− 27.00 ± 1.20^d^	− 5.33 ± 1.33^e^
Mean PR (%)	16.44	28.61	56.83	0.00	98.33
Repellency Class	Nonrepellent	II	III	Nonrepellent	V
Concentration of *G. sulphureus* extract (mg/ml)					
5	10.67 ± 1.33^a^	32.00 ± 1.15^b^	64.67 ± 1.76^c^	32.00 ± 2.00^d^	44.00 ± 2.31^e^
10	− 5.00 ± 1.53^a^	52.67 ± 1.76^ab^	71.33 ± 0.67^b^	82.00 ± 3.06^c^	36.67 ± 3.33^d^
15	− 24.00 ± 2.31^a^	55.33 ± 0.67^b^	78.67 ± 1.33^c^	87.00 ± 1.00^d^	6.00 ± 1.15^e^
20	− 21.33 ± 1.33^a^	42.00 ± 1.15^b^	64.33 ± 2.33^c^	89.67 ± 1.20^d^	71.00 ± 1.00^e^
25	− 24.00 ± 4.00^a^	26.67 ± 1.33^b^	66.00 ± 2.00^c^	84.00 ± 0.00^d^	66.67 ± 3.53^e^
30	− 31.33 ± 0.67^a^	34.00 ± 1.15^b^	67.00 ± 1.00^c^	98.67 ± 1.33^d^	− 5.33 ± 1.33^e^
Mean PR (%)	0.00	40.33	68.89	78.56	98.33
Repellency Class	Nonrepellent	III	IV	IV	V

Different letters in the same row indicate a significant difference (*p* < 0.05, Duncan’s test).

Mean PR = mean percentage of repellency. Mean of three replicates (n = 30 termites per replicate).

Repellency class = class 0 (> 0.01 to < 0.1%), nonrepellent; class I (0.1–20%), very weakly repellent; class II (20.1–40%), moderately repellent; class III (40.1–60%), averagely repellency; class IV (60.1–80%), fairly repellent; class V (80.1–100%), very repellent.

The results for *M. carbonarius* defense fluid at 1, 5, and 15 mg/ml showed significantly increasing repellency for 25 days (Table 3). However, from day 25 until day 30, the 1 and 5 mg/ml extracts showed a decline in repellence activities. In contrast, the highest concentration of *M. carbonarius* extract showed the opposite repellent activity trend, with 14.67% repellency at day 1 to no repellency at day 30 (− 27.00%). The percentage repellency for *G. sulphureus* extracts is also shown in Table 3. The percentage repellency of the 1, 5, and 15 mg/ml extracts increased at the beginning of the experiment and peaked after 20 days, and the extracts at 20 mg/ml showed increasing repellent activity. The highest performance (98.67%$$)$$ was observed after 30 days. At 1 mg/ml, the extracts showed repellency for only five days, after which no further activity was observed.

### Insecticidal effect of both extracts on termites

The LC_50_ results showed a significant effect on termites, and no insecticidal activity was recorded against *P. americana.* As observed in Table 4, the LC_50_ of the *G. sulphureus* extract was 16.917 mg/ml, which was lower than that of the *M. carbonarius* extract. This result may be related to the repellent activity of *M. gilvus*. The feed weight after mortality-inducing treatment of *M. gilvus* are presented in Table 5. The weight of the filter paper that was treated with *M. carbonarius* extract at 1 mg/ml increased from 0.024 g and decreased gradually. At 20 mg/ml, the feed weight increased, in contrast with the repellent activity of the *M. carbonarius* extract. Likewise, the mean feeding of *P. americana* on filter paper showed a decreasing trend from 0.075 g to 0.015 g as the concentration of the *G. sulphureus* extract increased.

**Table 4:** Half-lethal dose concentration (LC_50_) of the two different extracts against *Macrotermes gilvus.*

Treatment	n	*p*	LC_50_ (mg/ml)	Upper and lower boundaries
*M. carbonarius* extract	90	0.039	18.875	17.910–19.627
*G. sulphureus* extract	90	0.042	16.917	15.554–20.39

### Insecticidal efficiency of the extracts against termites

An increased percentage of termite mortality per day was detected when the concentrations of the extract increased (Table 6). However, termite mortality with *M. carbonarius* extracts at 15 and 20 mg/ml was different from that at other concentrations. A significant pattern of toxicity was observed for the *G. sulphureus* extract (Table 6). Extracts at all concentrations showed toxicity from day 3 until day 7, with 20 mg/ml causing the highest mortality. Both extracts showed the highest mortality on days 4 and 5 after treatments were administered. In addition, the *G. sulphureus* extract showed high mortality on days 4 and 5 compared to the *M. carbonarius* extract.

**Table 5:** Weight of feed consumed by *Macrotermes gilvus* after mortality-inducing treatment with the two types of extracts.

Concentration (mg/ml)	Mean feeding on filter paper (g)	
	Type of defense fluid extract	
	*M. carbonarius*	*G. sulphureus*
1	0.024 ± 0.002	0.075 ± 0.025
5	0.016 ± 0.001	0.028 ± 0.001
15	0.015 ± 0.001	0.021 ± 0.001
20	0.103 ± 0.002	0.015 ± 0.002

### Compound profiling of both extracts

**Figure 1:**
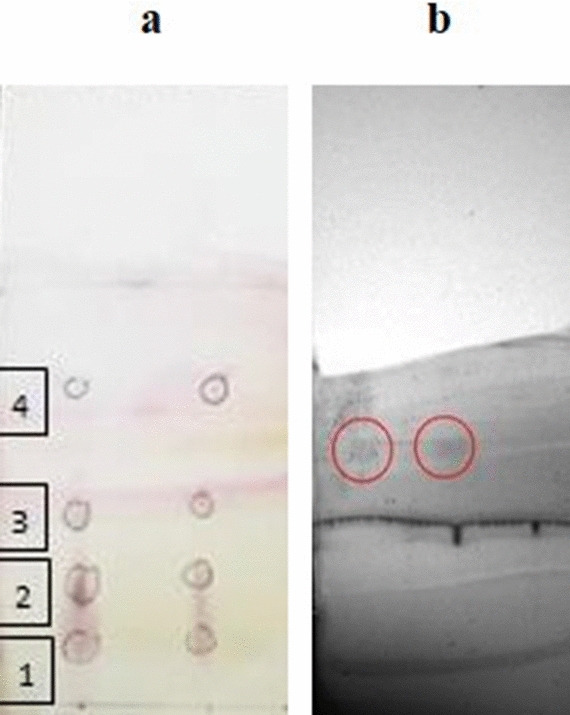
Thin layer chromatography (TLC) result of the *M. carbonarius* defense fluid extract shows four different compounds in plate **a** and the standard band (red circle) in plate **b**.

Four compounds from the *M. carbonarius* extract were obtained with retention factors of 0.11, 0.27, 0.42, and 0.70 by TLC (Fig. 1, Table 7). One of the compounds in band 4 was identified as lauric acid methyl ester after comparison with a standard in plate b. Thus, the results suggest that at least four compounds are responsible for the repellent and insecticidal activity of the extract against termites and cockroaches. A total of six major compounds from *M. carbonarius* were recognized using GC–MS (Table 8). The compound with the highest concentration was octadecanoic acid (20.95 mg/l), and that with the lowest concentration was lauric acid methyl ester (0.76 mg/l).

**Table 6:** Mean mortality percentage for seven days using *Macrotermes carbonarius* and *Globitermes sulphureus* extracts against *Macrotermes gilvus* at five different concentrations.

Day	Percentage of mortality per day (%)				
	0	1	5	15	20
**Concentration of M. carbonarius extract (mg/ml)**					
1	0.00 ± 0.00^a^	0.00 ± 0.00^b^	0.00 ± 0.00^c^	0.00 ± 0.00^d^	4.44 ± 0.43^e^
2	0.00 ± 0.00^a^	0.00 ± 0.00^b^	0.00 ± 0.00^c^	3.33 ± 0.11^d^	6.67 ± 0.29^e^
3	1.11 ± 0.37^a^	1.11 ± 0.04^b^	2.22 ± 0.07^c^	1.11 ± 0.04^d^	7.78 ± 0.08^e^
4	1.11 ± 0.37^a^	1.11 ± 0.35^b^	1.11 ± 0.04^c^	2.22 ± 0.07^d^	22.22 ± 0.07^e^
5	0.00 ± 0.00^a^	1.11 ± 0.04^b^	3.33 ± 0.11^c^	8.89 ± 0.40^d^	23.33 ± 0.15^e^
6	0.00 ± 0.00^a^	1.11 ± 0.04^b^	2.22 ± 0.07^c^	8.89 ± 0.40^d^	13.33 ± 0.11^e^
7	0.00 ± 0.00^a^	1.11 ± 0.00^b^	0.00 ± 0.00^c^	6.67 ± 0.11^d^	11.11 ± 0.04^e^
**Concentration of G. sulphureus extract (mg/ml)**					
1	0.00 ± 0.00^a^	1.11 ± 0.33^b^	0.00 ± 0.00^c^	0.00 ± 0.00^d^	2.22 ± 0.33^e^
2	0.00 ± 0.00^ab^	3.33 ± 0.58^b^	8.89 ± 1.46^c^	1.11 ± 0.33^d^	4.44 ± 0.33^e^
3	1.11 ± 0.33^ab^	8.89 ± 0.67^b^	8.89 ± 0.33^c^	15.56 ± 1.20^cd^	7.78 ± 0.67^e^
4	1.11 ± 0.33^ab^	5.56 ± 0.33^b^	11.11 ± 0.67^c^	17.78 ± 1.20^d^	24.44 ± 0.67^de^
5	0.00 ± 0.00^ab^	3.33 ± 1.00^bc^	4.44 ± 0.67^cd^	17.78 ± 2.40^d^	28.89 ± 2.40^de^
6	0.00 ± 0.00^a^	0.00 ± 0.00^b^	0.00 ± 0.00^c^	4.44 ± 0.89^d^	14.44 ± 0.33^e^
7	0.00 ± 0.00^a^	0.00 ± 0.00^b^	0.00 ± 0.00^c^	6.67 ± 0.00^d^	11.11 ± 2.00^e^

Different letters in the same row indicate a significant difference (*p* < 0.05, Duncan’s test).

**Table 7:** Retention factor determined by TLC for *Macrotermes carbonarius* defense fluid, calculated from plate a and compared to the retention factor of the standard in plate b.

Compound	Plate	Band	Retention factor (Rf)	Compounds identified
–	a	1	0.11	Unknown
–	a	2	0.27	Unknown
–	a	3	0.42	Unknown
Lauric acid methyl ester	a (Extract)	4	0.70	Pentadecanoic acid
	b (Standard)	1	0.69	

**Figure 2:**
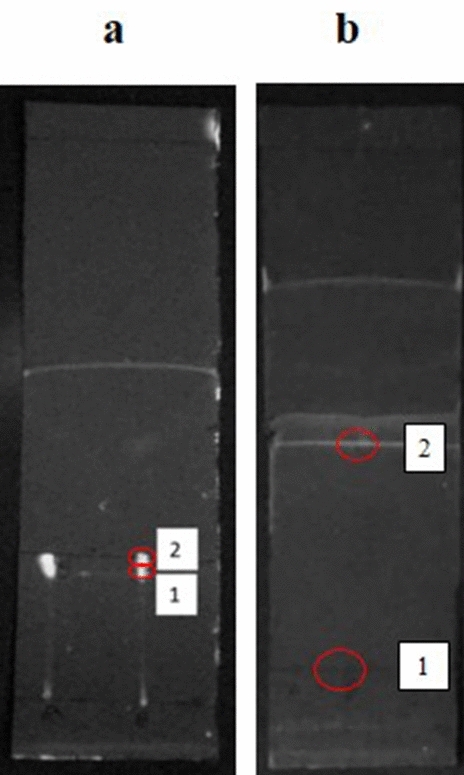
Thin layer chromatography (TLC) results of *G. sulphureus* defense fluid extract in plate **a** and standard band in plate **b** under 254 nm UV light in a UV Viewing Chamber (UVITEC, England, United Kingdom). The red circle indicates a band obtained on both plates.

Figure 2 and Table 9 demonstrate the compound separation on thin layer chromatography (TLC) for the *G. sulphureus* extract. In this experiment, two compounds were spotted on plate a after the extract was developed. The compounds were observed at retention factors of 0.47 and 0.43, and one of the compounds was identified as pentadecanoic acid.

**Table 8:** Compound profile of *Macrotermes carbonarius* defense fluid determined by GC–MS, with retention time, similarity, concentration and compound activity.

Peak	Retention time (RT)	Compound name	Similarity	Concentration in 100 mg/l
1	6.908	Furanone	95	9.76
2	9.781	Lauric acid methyl ester	91	0.76
3	11.860	Hydroquinone	91	19.61
4	12.490	Pentadecanoic acid	94	15.0
6	13.476	*Cis*-Vaccenic acid	84	11.16
7	13.583	Octadecanoic acid	90	20.95

Eight compounds were discovered in this study by GC–MS analysis (Table 10). The highest concentration was recorded for octadecanoic acid (16.24 mg/l), and methyl stearate (0.67 mg/l) was the compound with the lowest concentration.

**Table 9:** Retention factor determined by TLC for *Globitermes sulphureus* defense fluid, calculated from plate a and compared to the retention factor of the standard in plate b.

Compound	Plate	Band	Retention factor (Rf)	Compounds identified
Tridecane	a (Extract)	1	0.45	Unknown
	b (Standard)	1	0.30	
Pentadecanoic acid	a (Extract)	2	0.47	Pentadecanoic acid
	b (Standard)	2	0.50

**Table 10:** Compound profile of *Globitermes sulphureus* defense fluid determined by GC–MS, with retention time, similarity, concentration and compound activity.

Peak	Retention time (RT)	Compound name	Similarity	Concentration (mg/l) in 78 mg/l
1	8.180	Tridecane	95	1.87
2	9.785	Phenol, 3,5-bis(1,1-dimethylethyl)-	91	1.62
4	12.304	Hexadecanoic acid, methyl ester	91	6.14
6	12.493	Pentadecanoic acid	94	11.32
7	13.392	Methyl stearate	88	0.63
8	13.586	Octadecanoic acid	90	16.24

## Discussion

The yield of defense fluid was influenced by insect size and method of defense against threats. *Globitermes sulphureus* is smaller in size and does not have dimorphic soldiers that act as major and minor soldiers during defense, unlike *M. carbonarius*. Thus, a high volume of defense fluid production is crucial for the chemical defense mechanism of *G. sulphureus*. Small soldiers such as nasutoids usually rely mainly on the chemical weapons produced by their large front gland, whereas large-jawed soldiers produce less defensive secretion^6,30,31^. Termites also produce significantly more defense fluid than other social insects, such as ants, due to differences in the mechanism of action^32,33^.

These chemical cues or pheromones are required for various purposes, such as for the recognition of biological processes and caste identification. The presence of *M. carbonarius* and *G. sulphureus* extracts enabled *M. gilvus* to recognize them as isolated chemical cues other than their own. In nature, the presence of different cuticular compounds in one-piece nesting termites causes avoidance, and they become aggressive^34^. Specific species, such as *M. gilvus,* respond to the situation with some level of alarm or avoidance even at a different distances from their colonies^35^. In the present study, the presence of active compounds such as quinone in the *M. carbonarius* extract could also influence repellency at the highest concentration^6^.

The *G. sulphureus* extracts were also effective at lower concentrations. This outcome is contrary to the study of Benth plants, in which treatment with *Derris elliptica* (Fabales: Fabaceae) extract at 5 mg/ml led to a 6.6% reduction in *M. gilvus* attack after seven days^36^. This finding suggested that in addition to existing botanical insecticides, termite defense fluid has potential applications as a natural repellent. The efficiency of *G. sulphureus* extract is likely related to the defense mechanism of the carpenter ant *Camponotus* spp. (Hymenoptera: Formicidae), which is similar to *G. sulphureus.* It releases polyacetate-derived aromatic compounds during autothysis to kill or repel enemies^37,38^. It is therefore likely that the repellent activity of *G. sulphureus* defense fluid is the same as that of *Camponotus* spp.

The study shows that both extracts tested in this study share the same trend of repellency activity, but the longevity and efficiency were concentration-dependent for both extracts. The extracts from this study showed the same trend for the dose-dependent behavioral response when insects were exposed to pheromones^39^. Our findings on *M. gilvus* repellency at least hint that the defense fluid has the ability to act as a natural repellent, as the species exhibits the lowest susceptibility to baiting or other chemical pesticides^40^.

Overall, the repellent activities of *M. carbonarius* and *G. sulphureus* defense fluid against the American cockroach were lower than those against *M. gilvus*. This difference caused by the differences in function, method, and reception in terms of chemical communication for cockroaches and termites. Chemical communication mechanisms of cockroaches involve mainly sex pheromones and aggregation^41^. Furthermore, cockroach protection mechanisms are much more advanced in the presence of nocifensive behavior that is stimulated by sensory stimuli for potential injury to insects^42^. This finding explains the low response of cockroaches to the repellence. This condition may also be due to the differences in the interpretation of chemical cues between both species. For instance, male moths are more strongly attracted to pheromone traps baited with a blend of synthetic pheromones mixed with some plant-related volatiles than to pheromones alone^43^. The same response was observed in a study on *P. americana* antennae^44^.

Generally, the repellent activity of *M. carbonarius* extract was influenced by extract concentration. However, at the highest concentration, no repellent activity was observed due to the occurrence of odor generalization. This is because components of alarm pheromones cause more generalization than botanical compounds in insects with olfactory receptors^45^. At low concentrations, repellent activity is present due to low stimulus loads, and olfactory receptors (ORs) are more narrowly changed^46–48^. This explains the response of the American cockroach toward *M. carbonarius* extract*.* In addition, the *G. sulphureus* extract exhibited better performance than the *M. carbonarius* extract due to the differences in specific compounds in the *G. sulphureus* extract. This can be observed from the stable repellent activity of the extract at 20 mg/ml. The efficiency of *G. sulphureus* defense fluid extract as a repellent against the American cockroach for 30 days makes it a promising alternative as an insecticide.

*Macrotermes carbonarius* extract has a higher LC_50_ than *G. sulphureus* extract. This may be explained by the presence of toxic compounds such as quinone. This compound has been reported as a toxic, ubiquitous defensive compound found in arthropods such as the red flour beetle *Tribolium castaneum* (Coleoptera: Tenebrionidae)^49–53^. The reason for the low LC_50_ of *G. sulphureus* defense fluid is its aggressive and toxic effects. When threatened, this insect ruptures a large gland and release the thick, yellow fluid that entangles ants or other insects that are trying to invade their nest^54,55^. The defense fluids of other species, such as *Neocapritermes taracua* (Termitidae: Termitinae) and *Ruptitermes*, also showed to repellent and death-inducing effects^32,56^.

In this study, the weight of filter paper consumed by *M. gilvus* was connected to the percentage of repellency for both extracts. On the other hand, the highest concentration of *M. caronarius* showed high attraction of termites, as determined by filter paper consumption. This result is consistent with those of previous studies. The presence of hydroquinone stimulated termite feeding, as in the Darwin termite, *Mastotermes darwiniensis* (Blattodea: Mastotermitidae), and *Coptotermes acinaciformis* (Blattodea: Rhinotermitidae), which consumed significantly more wood in the presence of hydroquinone^57^. Another possible explanation for this is that the same substance can act as a repellent or attractant depending on the conditions used in the bioassay^58,59^. These findings may help us to understand the importance of concentration and the compound involved in the food preferences of *M. gilvus.*

For mortality of termites per day, at high concentrations of *M. carbonarius* extracts, the mortality corresponded to the highest consumption of filter paper (0.103 g). Continuous feeding caused a sudden increase in termite mortality. Thus, the compound identity, concentration and behavioral context of semiochemicals need to be taken into account as tools for insect control^60^. The *G. sulphureus* extract had a mortality effect that was associated with the percentage repellency. The toxicity was not only caused by ingestion but also starvation due to the high repellency effect. This result is consistent with studies conducted by other researchers^23^. described that the strong repellency of a toxic plant led to slow death of *G. sulphureus *and *Coptotermes gestroi* due to starvation, while close contact with the extract led to the termites becoming disoriented and eventually dying. Therefore, as both studies used extracts to study the toxicity, the similarity is reasonable.

The results highlighted the compounds from the two species. In this study, hydroquinone was shown to be related to the repellent activity (Table 8). Another study showed that hydroquinone isolated from Formosan subterranean termites, *Coptotermes formansus*, repelled the same species, and no increase in tunneling activity was observed in the sand tested^61^. In contrast, in this study, hydroquinone had an alternate function as an attractant at certain concentrations, in addition to its insecticidal effect. This compound plays a role as a phagostimulant that attracts termites such as *M. gilvus* to the feeding site^52,62^. The function of this compound is consistent with a previous study, which may explain the sudden increase in the consumption of filter paper by *M. gilvus* at a 20 mg/ml extract concentration. The compound had the second-highest concentration in the extract, and advanced testing is required for further confirmation. The insecticidal activity was also contributed to by other compounds with insecticidal properties, which were observed in a previous study^63^. Other compounds, namely, pentadecanoic acid and furanone, were believed to be responsible for the repellency activity, as shown in a previous study, but no detailed investigation was performed in this study^64,65^. These compounds were believed to contribute to the LC_50_.

Repellent constituents of *G. sulphureus* are influenced by pentadecanoic acid, hexadecanoic acid methyl ester and tridecane^66^. Tridecane is widely acknowledged since it was included in integrated pest management as a semiochemical pesticide for the same species^67^. Phenol and hexadecanoic acid methyl ester have dual functions, as repellent and insecticide, which is consistent with the previous study. This compound may also contribute to the efficiency of the defense fluid extract as a repellent and insecticide, but further detailed investigations are needed^63,68–71^. The insecticidal activity of this extract was contributed to by octadecanoic acid and stearic acid, leading to a low LC_50_ of this extract (16.92 mg/ml), as inferred in a previous study. A study described in^72^ proved that a formulation of fatty acid methyl ester (FAME) that consists of methyl stearate showed larvicidal activity against *C. quinquefasciatus*. The previous study showed that the insecticidal activity of this compound occurred through ingestion, which is in consistent with and validated the result of the present study.

Our results demonstrated that the similarity and differences of the compounds compared to a previous study give rise to different effects of repellency and toxicity. The different functions of the two extracts led to different results and performances. Both defense fluid extracts showed repellent and insecticidal effects. These extracts were also identified as termiticides. However, *G. sulphureus* defense fluid has more potential as a natural repellent and insecticide than *M. carbonarius* defense fluid. Even though the chemical identities of individual extract components were determined, their specific insecticidal and repellent activities await determination. This may be considered a promising aspect of new effective potential repellents and insecticides that use termite chemical communication. Further work is certainly required to unravel the complexities of the synergistic activity of the compounds, the behavioral context, and field application.
